# Supplementary material for: Single-cell and spatiotemporal transcriptomic profiling of brain immune infiltration following Venezuelan equine encephalitis virus infection
Source: Front Immunol. 2024 Dec 19;15:1497839. doi: 10.3389/fimmu.2024.1497839 (PMC11693676; doi:10.3389/fimmu.2024.1497839)
Supplement: Supplementary file 1 [file DataSheet1.docx]

**Supplementary Fig. 1**


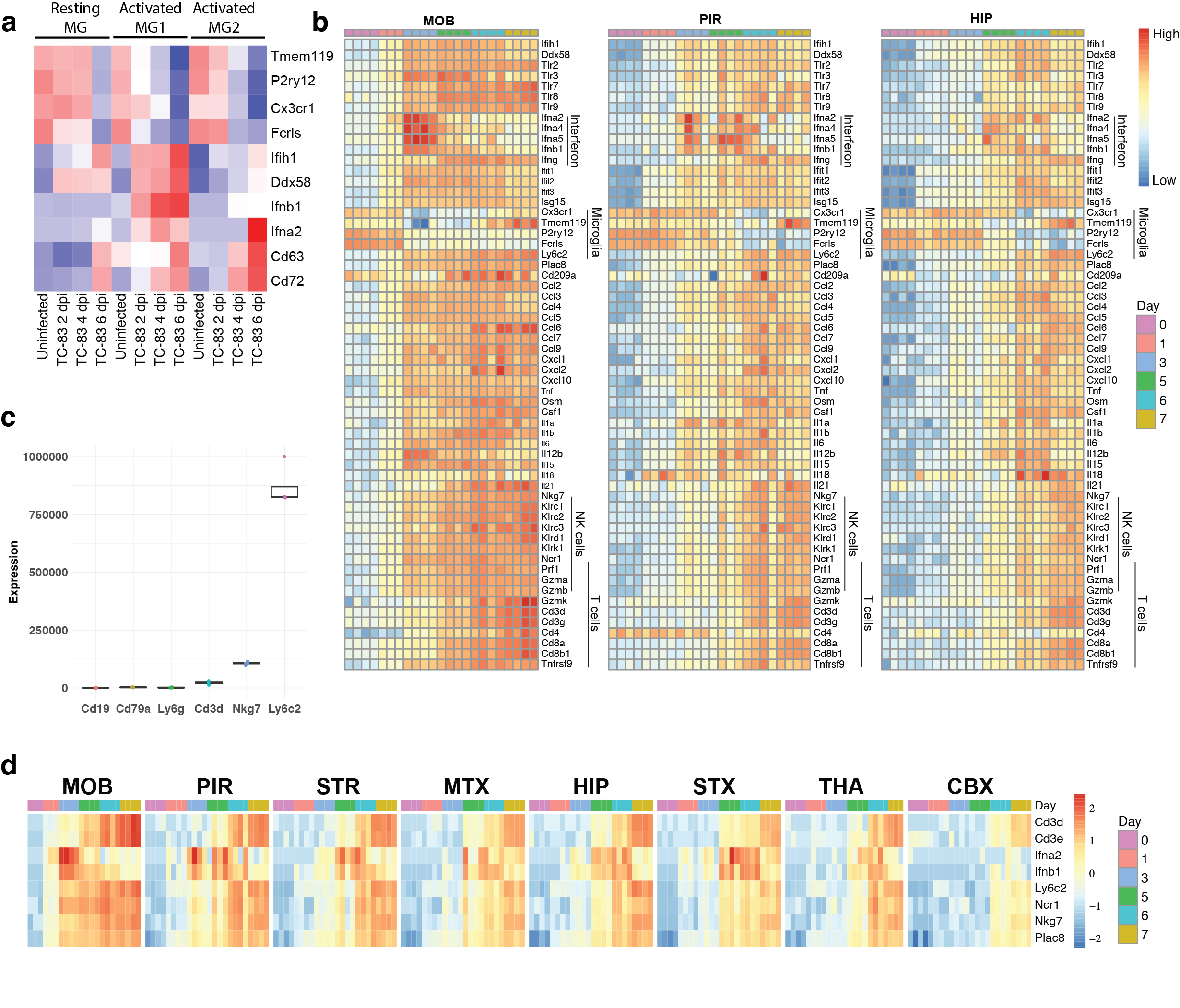


**Supplementary Fig. 2**


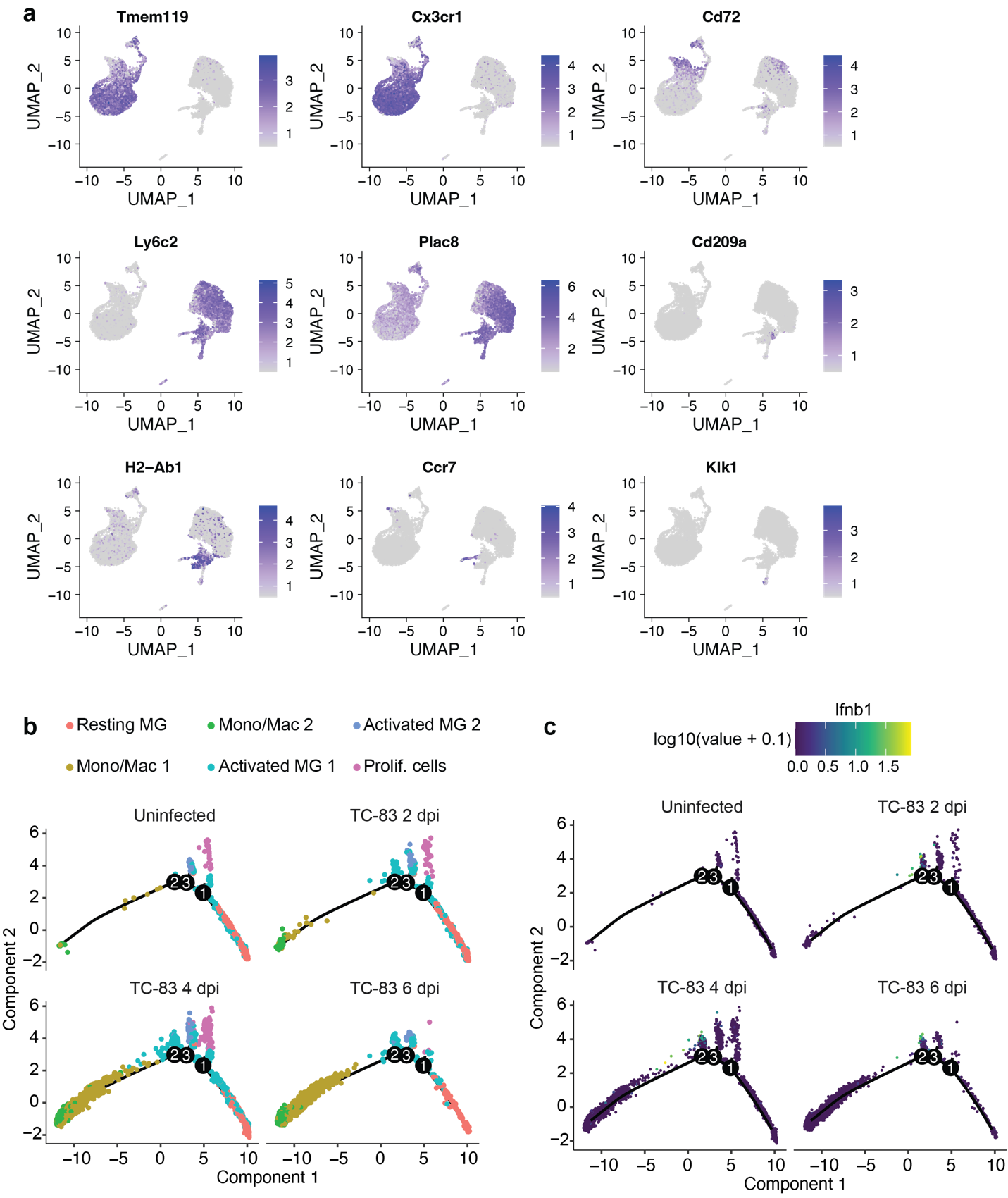


**Supplementary Fig. 3**

**
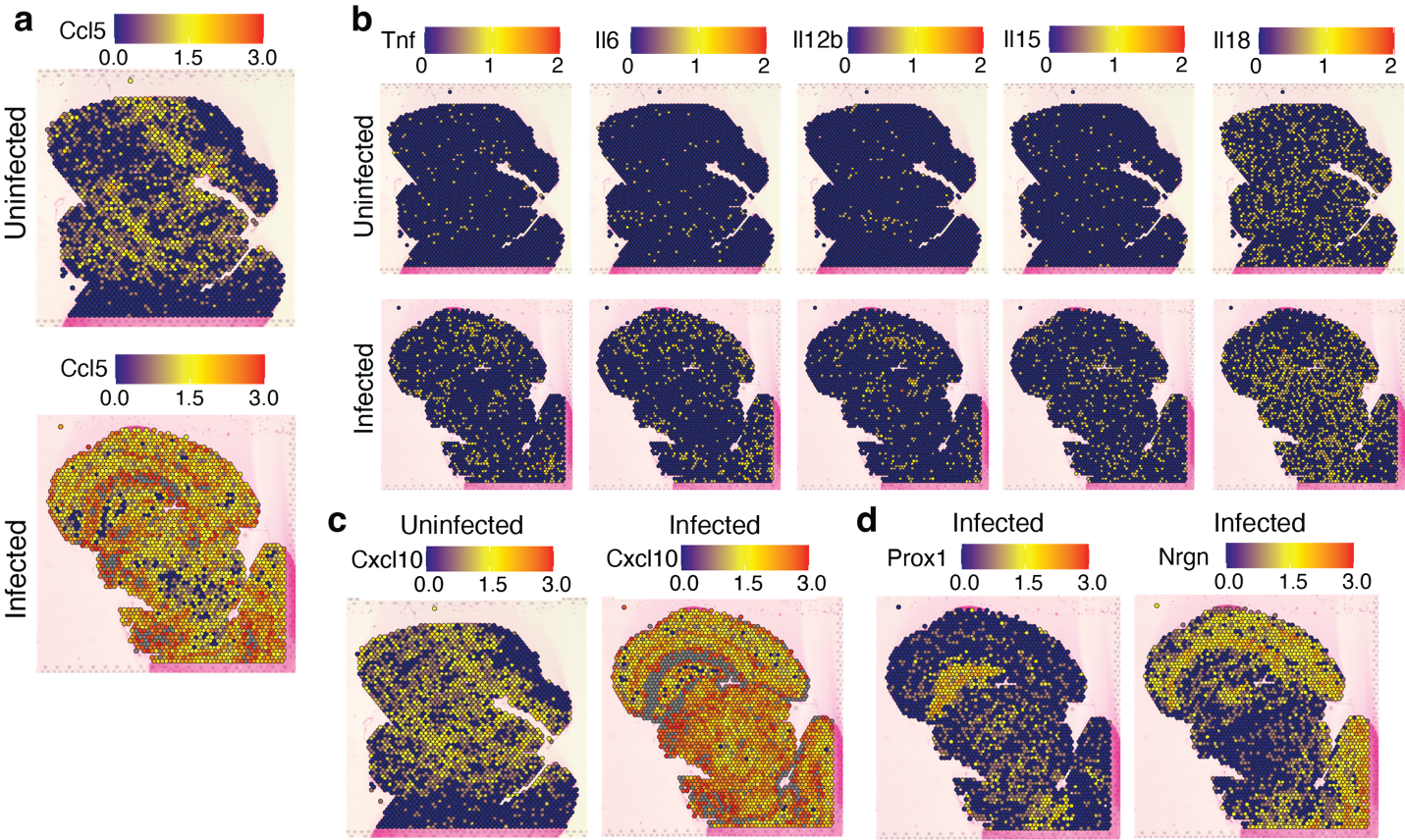
**

**Supplementary Fig. 4**

**
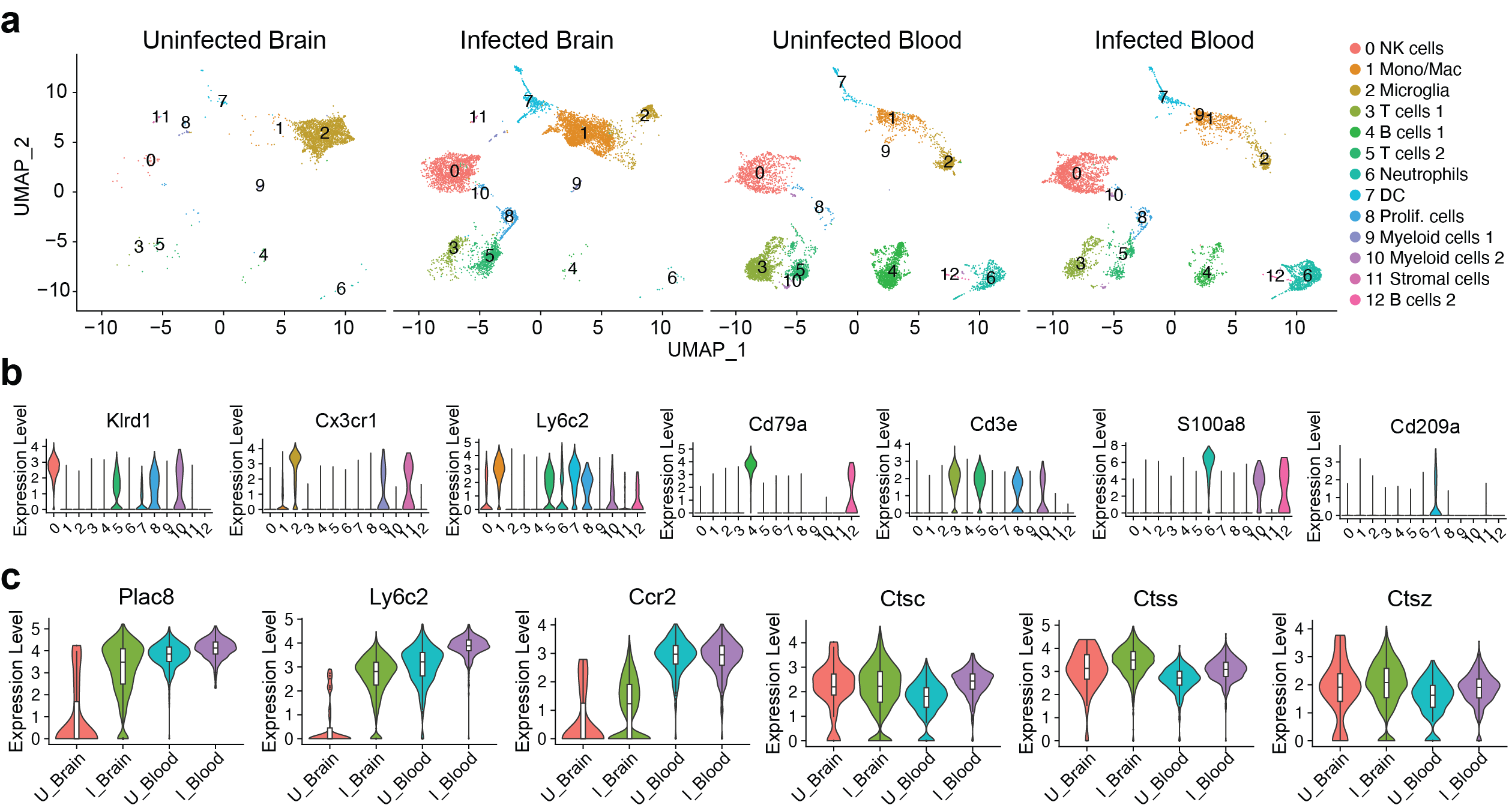
**

**Supplementary Figure Legends**

**Supplementary Fig. 1: Temporal and spatial gene expression of immune cells in the brain during VEEV infection.** **a** Heatmap showing expression of select antiviral response genes in microglial subclusters across experimental groups in this study. Re-analysis of a bulk RNA sequencing data set previously published by Williams et al. [20] was performed to generate heatmaps showing expression of key immune genes over time in the main olfactory bulb (MOB), piriform cortex (PIR), and hippocampus (HIP) (**b**), a boxplot showing overall expression of select cell identity markers in the MOB at 6 dpi (**c**), and heatmaps showing expression of select genes associated to prominent infiltrating populations in the MOB, PIR, striatum (STR), motor cortex (MTX), HIP, sensory cortex (STX), thalamus (THA), and cerebral cortex (CBX) over time (**d**).

**Supplementary Fig. 2: Gene expression in myeloid subclusters in the brain during VEEV infection.** **a** Feature plots show the differential expression of select genes in myeloid subclusters detected in the brain. Trajectory analysis of myeloid subclusters, colored by identity (**b**) or *Ifnb1* expression (**c**).

**Supplementary Fig. 3: Spatial profiles of myeloid gene expression in the brain during VEEV infection.** Distribution of gene expression of *Ccl5* (**a**), *Tnf* and interleukins (**b**), and *Cxcl10* (**c**) is shown in uninfected versus infected brains at 6 dpi as determined by spatial transcriptomic analysis. (**d**) Distribution of expression of region-specific markers *Prox1* (HPF) and *Nrgn* (CBX) in infected brains.

**Supplementary Fig. 4: Transcriptional profiling of brain immune cells and PBMCs during VEEV infection. a** UMAP visualization cell clusters generated from scRNA-seq of uninfected or VEEV-infected blood or brain. **b** Violin plots showing expression of identifying markers across cell clusters. **c** Expression of select genes in mono/mac population of uninfected or VEEV-infected brains or blood.
